# Risk factors and outcome associated with coinfection with carbapenem-resistant *Klebsiella pneumoniae* and carbapenem-resistant *Pseudomonas aeruginosa* or *Acinetobacter baumanii:* a descriptive analysis

**DOI:** 10.3389/fcimb.2023.1231740

**Published:** 2023-10-16

**Authors:** Anthony Sophonsri, Corey Kelsom, Mimi Lou, Paul Nieberg, Annie Wong-Beringer

**Affiliations:** ^1^ Alfred E. Mann School of Pharmacy and Pharmaceutical Sciences, University of Southern California, Los Angeles, CA, United States; ^2^ Department of Pharmacy, Huntington Hospital, Pasadena, CA, United States; ^3^ Department of Infectious Diseases Medicine, Huntington Hospital, Pasadena, CA, United States

**Keywords:** carbapenem resistance, multidrug resistance, coinfection, *Klebsiella pneumoniae*, *Pseudomonas aeruginosa*, *Acinetobacter baumanii*

## Abstract

**Background:**

Nearly 30% of patients infected with carbapenem-resistant *Klebsiella pneumoniae* (CRKP) were previously shown to be coinfected with carbapenem-resistant *Pseudomonas aeruginosa* (CRPA) or *Acinetobacter baumannii* (CRAB). Infections caused by multiple carbapenem-resistant pathogens present significant challenge to infection control and therapeutic management. The study objective was to identify risk factors for acquisition of multiple carbapenem-resistant pathogens and associated outcomes.

**Methods:**

A descriptive analysis of adults infected with either CRKP alone or coinfected with CRPA or CRAB was performed. Patient groups were compared on demographics, clinical characteristics, treatment, and outcome.

**Results:**

86 patients with CRKP monoinfection and 60 patients with coinfections were evaluated. Respiratory tract was the predominant infection site for coinfected patients involving mostly CRPA whereas urinary tract was the primary site for CRKP-only group. More coinfected patients were severely debilitated, had prior carbapenem exposure (37% vs 13%, p<0.001) and history of pneumonia in the past year (67% vs 41%, p<0.01). More coinfected patients required direct ICU admission (45% vs 27%, p=0.02) and had prolonged length of stay (median 15 vs 10 days, p<0.01) than the CRKP-only group but mortality rates (18% vs 16%) were similar.

**Conclusions:**

CRKP coinfection with another carbapenem-resistant pathogen adds significant morbidity and healthcare burden overall. Empiric therapy with reliable activity against both CRKP and carbapenem-resistant *Pseudomonas aeruginosa* may be prudent for at risk patients with pneumonia.

## Background

Patients colonized or infected with carbapenem-resistant Enterobacterales (CRE), namely carbapenem-resistant *Klebsiella pneumoniae* (CRKP), may become simultaneously or subsequently co-colonized or coinfected with other carbapenem-resistant Enterobacterales as well as non-fermenting gram-negative pathogens such as carbapenem-resistant *Pseudomonas aeruginosa* (CRPA) or *Acinetobacter baumanii* (CRAB) (Marchaim et al., 2012). Coinfection with multiple carbapenem-resistant pathogens poses significant challenge for infection control and therapeutic management. A study in the U.S. involving 5 hospitals in 4 states found that 8.9% of patients were co-colonized with at least 2 different species of carbapenem-resistant Enterobacterales (Adediran et al., 2020). Others have shown that predictors of co-colonization (defined as CRE-positive cultures with CRPA or CRAB within a 7-day time period) were recent stay at a long-term care facility, previous antimicrobial therapy targeting Gram-positive organisms and high Charlson Comorbidity Index score (Marchaim et al., 2012). Increased 90-day mortality was also observed in co-colonized patients in that study, raising the possibility that the presence of CRPA/CRAB contributes to a higher severity of illness and poor outcome.

Specifically for CRKP coinfection with carbapenem-resistant non-fermenting Gram-negative pathogens, there is limited data on the risk factors and associated outcome to date. A single-center study conducted in China focused on coinfection with CRKP and CRAB and found that coinfection was associated with significantly prolonged hospital stays and higher mortality rate compared to cohorts infected with either pathogen alone (Lv et al., 2022). However, the relationship between infection type and/or delayed start of effective therapy and outcome was not evaluated. Our group has previously shown that patients hospitalized for CRKP non-bacteremic infections such as pneumonia or urinary tract infection experienced both prolonged hospital stays and alarmingly high 30-day readmission rates of 33% (Ny et al., 2015). Notably, 27% had coinfection with other carbapenem-resistant bacteria including CRPA and CRAB (Ny et al., 2015). Herein, we sought to identify risk factors for coinfection with either CRPA or CRAB in CRKP-infected patients and assess the impact of coinfection on outcomes.

## Methods

This was a retrospective descriptive analysis conducted between 2010 and 2020 at a 619-bed community-teaching hospital. The study was approved by the hospital’s Institutional Review Board (Advarra IRB: Pro00036497); informed consent was waived. Procedures followed were in accordance with the ethical standards of the institutional IRB and with the Helsinki Declaration of 1975. Medical records of adult patients hospitalized with culture-documented CRKP from any source (blood, respiratory, urine, wound, other) were reviewed to identify patients with concurrent isolation of CRPA and/or CRAB from any source. Patients with monomicrobial CRKP infection were classified as ‘CRKP-only’ while those also infected with either CRPA or CRAB were classified as ‘coinfected’. Patients were excluded if they had the following: 1) positive cultures from an emergency department encounter without inpatient admission, 2) only carbapenem-susceptible *Pseudomonas aeruginosa* or *Acinetobacter baumanii* was present during the same hospital admission, 3) positive cultures without evidence of infection or without the need for treatment for both CRKP and CRPA, or 4) medical records were unavailable for review.

Medical records were reviewed to obtain demographic, laboratory, radiographic, microbiologic and other pertinent clinical information, as well as details of antimicrobial therapy (drug, dose, duration) during admission and in the 90 days prior to CRKP, CRPA or CRAB isolation. All data were collected for the index hospital admission during which CRKP-only or CRKP plus CRPA or CRAB were isolated. Patient-specific information was extracted and recorded in REDCap, a HIPAA-compliant secured electronic database hosted at the University of Southern California (Harris et al., 2009).

### Study definitions

Coinfection with CRKP and CRPA or CRAB was defined as isolation of CRKP and CRPA or CRAB within 7 days along with a documented diagnosis of infection on the basis of clinical and/or diagnostic signs and symptoms in the medical record. An invasive infection was defined as either bacteremia or pneumonia, while non-invasive infections included urinary tract infections and skin and soft tissue infections. Carbapenem resistance was defined by a minimum inhibitory concentration (MIC) >1 mg/L for meropenem or imipenem and > 0.5 mg/L for ertapenem among *K. pneumoniae* isolates and a MIC >2 mg/mL for meropenem or imipenem among *P. aeruginosa* and A*. baumanii* isolates according to the Clinical and Laboratory Standards Institute (CLSI) guidelines, as determined using the BD Phoenix Automated Microbiology System (BD Diagnostic Systems, Sparks, MD) or by a positive modified Hodge test. Therapy was deemed effective if the carbapenem-resistant organism was susceptible *in vitro* to at least one agent in the prescribed regimen. Functional status of patients at time of admission and discharge was assessed using the Katz index, which measures the patient’s ability to perform activities of daily living independently (Katz et al., 1970). The index ranks adequacy of performance in the six functions of bathing, dressing, toileting, transferring, continence and feeding, with a score of six indicating full function and a score of two or less indicating severe functional impairment. Clinical stability was defined as systolic blood pressure ≥90 mm Hg, respiratory rate ≤24 breaths per minute, temperature <38°C, oxygen saturation ≥90% on room air, off mechanical ventilation, and return to baseline mental status (Halm et al., 1998).

### Statistical analysis

CRKP-only and coinfection study groups were compared on demographics, clinical characteristics, and outcome measures: time to clinical stability, length of stay, discharge disposition, functional status at time of discharge, readmission, and in-hospital mortality. Chi-square or Fisher’s exact test was performed on categorical variables and Mann-Whitney U or student’s t-test was performed on continuous variables where appropriate. Multivariable logistic regression analyses were performed to identify risk factors associated with coinfection by using forward selection with the best Schwarz Bayes Criterion (SBC) model in high performance procedure. Age and sex were controlled in the model. Statistical analyses were performed using SAS version 9.4 (SAS Institute). A p-value of ≤0.05 denoted statistical significance.

## Results

### Baseline characteristics

A total of 146 patients met study inclusion criteria: 86 had CRKP monoinfection and 60 patients had coinfection with CRKP and CRPA or CRAB. Of those with coinfections, 75% (45/60) had CRKP and CRPA, 13% (8/60) had CRKP and CRAB while 11% (7/60) had all three pathogens (CRKP, CRPA and CRAB). The study population was predominantly elderly with a median age of 73 and 70 years in the CRKP-only and coinfected groups, respectively (**Table 1**). Most patients were admitted from a skilled nursing facility (SNF) or long-term care facility (LTCF) (71% CRKP-only and 82% coinfected), coinfected patients were significantly less likely to be admitted from home (8% vs 28%, p<0.0001). Nearly all coinfected patients (92%) lack the ability to perform activities of daily living compared to 60% of the CRKP-only group (p<0.0001) as measured by the Katz index score of zero; the main drivers for this observed difference between groups appeared to be continence and feeding. Almost twice as many coinfected patients were directly admitted to the ICU (45% vs 27%, p=0.02). While the majority of patients in both groups had prior healthcare exposure, more coinfected patients had a history of pneumonia in the past year (67% vs 41%, p<0.01) and more had documented antimicrobial exposure within 90 days (72% vs. 54%, p=0.03). Prior exposure to carbapenem, beta-lactam/beta-lactamase inhibitor, tigecycline, parenteral vancomycin, and metronidazole were significantly higher for the coinfected group (**Table 1**). Notably, a greater proportion of coinfected patients had a prior history of culture positive for ESBL-producing (27% vs 13%, p=0.03) and carbapenem-resistant organisms (17% vs 2%, p=0.004).

**Table 1:** Baseline patient characteristics.

	CRKP-Only n=86 (%)	Coinfected n=60 (%)	p-value
Age, median (IQR)	73 (61, 84)	70 (59, 80)	0.16
Gender, female	49 (57)	24 (40)	0.04
Residence prior to admission			<0.001
Home	24 (28)	5 (8)	
Skilled nursing /long term care facility	61 (71)	49 (82)	
Outside hospital	1 (1)	6 (10)	
Direct ICU admission	23 (27)	27 (45)	0.02
Charlson Comorbidity Index (CCI), median (IQR)	6 (5, 8)	6 (4, 8)	0.79
Katz score of zero at time of admission	52 (60)	55 (92)	<0.0001
Prior Healthcare Exposure			
Healthcare exposure in past year	75 (87)	58 (97)	0.07
History of infection in past year	66 (77)	51 (86)	0.15
Pneumonia	27 (41)	34 (67)	<0.01
Urinary tract infection	36 (55)	33 (65)	0.28
Skin and soft tissue infection	21 (32)	22 (43)	0.21
Bacteremia	10 (15)	14 (27)	0.10
C. difficile colitis	15 (23)	7 (14)	0.22
Osteomyelitis	8 (12)	3 (6)	0.34
Antimicrobial exposure in past 90 days	44 (54)	43 (72)	0.03
Carbapenem	11 (13)	22 (37)	<0.001
Beta-lactam/beta-lactamase inhibitor	14 (16)	18 (30)	0.05
Tigecycline	2 (2)	7 (12)	0.03
Vancomycin (intravenous)	11 (13)	20 (33)	0.003
Metronidazole	9 (10)	13 (22)	0.06
Systemic antifungal	7 (8)	11 (18)	0.07
Other*	21 (24)	24 (40)	0.04
History of ESBL-positive culture	11 (13)	16 (27)	0.03
History of carbapenem-resistant positive culture	2 (2)	10 (17)	0.004
Surgical procedure in past 30 days	6 (7)	5 (8)	0.76

*Other includes systemic antimicrobial agents where n<10 in each group: aminoglycosides, cefepime, colistin (intravenous), daptomycin, fluoroquinolones, linezolid.

### Microbiologic and clinical characteristics

Anatomic site from which the study organism was isolated differed between the two groups (**Table 2**). The predominant source of infection for the coinfected group was the respiratory tract compared to urine as the primary source in the CRKP-only group. Among the coinfected group, CRKP and CRPA or CRAB pairs were mostly (65%, 39/60) isolated from the same culture (**Table 2**). Among patients coinfected with CRPA, 71% (32/45) were cultured from the respiratory tract and one (2%) from the blood. Overall use of medical devices was greater in the coinfected than CRKP-only group (**Table 3**). The need for mechanical ventilation was nearly 3-fold higher in the coinfected compared to the CRKP-only group (73% vs 26%, p<0.0001) with a significantly prolonged duration of ventilation during hospitalization (median 14 days vs. 10 days, p=0.02). More coinfected patients also required chronic Foley catheter placement (68% vs. 49%, p=0.02); A similar trend was noted for feeding tube requirement (90% vs. 49% p<0.0001) and for a more prolonged duration (median 14 days vs. 10 days, p=0.004). Patients who were coinfected presented more severely ill than those with CRKP-only as more required ICU admission (60% vs. 31%, p<0.001).

**Table 2:** Culture Site Comparison between CRKP only and Coinfected groups.

Anatomic site of culture	CRKP-only, n=86 (%)		Coinfected, n=60 (%)	
	Single site, n=78	Multiple sites, n=8*	Same site, n=39	Different sites, n=21
Urine	58 (74%)	6 (37.5%)	9 (23%)	10 (24%)
Respiratory	12 (15%)	4 (25%)	24 (61%)	18 (43%)
Wound	3 (4%)	2 (12.5%)	3 (8%)	7 (17%)
Blood	2 (3%)	2 (12.5%)	1 (3%)	6 (14%)
Other	3 (4%)	2 (12.5%)	2 (5%)	1 (2%)

*Eight patients in CRKP only group had positive culture for CRKP grown from multiple body sites accounting for 16 positive cultures.

†21 co-infected patients had positive cultures for CRKP, CRPA or CRAB grown from multiple body sites accounting for 42 positive cultures.

### Antimicrobial utilization and concomitant medications

Empiric and directed antimicrobial therapy during admission were compared between the study groups. Notably, a higher proportion of coinfected patients were initiated on an effective empiric antimicrobial regimen than the CRKP-only group (40% vs 10%, p<0.0001), though the time to receipt of effective antimicrobial therapy was similar between the two groups at a median of 3 days (**Table 3**). The overall treatment duration was prolonged by 5 days (median 14 vs. 9 days, p<0.001) in the coinfected group compared to CRKP-only group. With respect to the antimicrobial agents prescribed, use of carbapenems (72% vs. 41%, p=0.0002) and agents active against Gram-positive pathogens (85% vs 63%, p=0.003) was significantly higher in the coinfected group; however, the use of agents with anti-anaerobic activity (i.e., metronidazole) did not differ between the two groups. Additionally, most patients received an acid-suppressing agent (e.g. proton pump inhibitors and histamine_2_ receptor antagonists) with a trend towards higher utilization in the coinfected group (92% vs 80%, p=0.06) and for a longer duration (14 days vs. 10 days, p=0.09) during hospitalization. Receipt of immunosuppressive therapy was found in about one third of the patients and did not differ between the study groups.

**Table 3:** Clinical Course and Management.

	CRKP-Only n=86 (%)	Coinfected n=60 (%)	p-value
Use of Medical Device			
Mechanical ventilation	22 (26)	44 (73)	<0.0001
Duration of mechanical ventilation, days median (IQR)	10.5 (4, 14)	14 (9, 25)	0.02
Central line insertion	30 (35)	34 (57)	<0.01
Duration of central line insertion, days median (IQR)	12 (5, 23)	13 (9, 27)	0.25
Chronic Foley catheter requirement	42 (49)	41 (68)	0.02
G-tube/NG-tube requirement	42 (49)	54 (90)	<0.0001
Duration of G-tube/NG-tube insertion, days median (IQR)	10 (4, 14)	14 (10, 22)	0.004
ICU Admission	27 (31)	36 (60)	<0.001
Duration of ICU stay, days, median (IQR)	6 (3, 14), n=27	8 (3.5, 13), n=36	0.41
Antibiotic Treatment			
Effective empiric therapy	9 (10)	24 (40)	<0.0001
Time to effective antimicrobial therapy, days, median (IQR)	3 (2, 4)	3 (3, 4)	0.14
Overall treatment duration, days, median (IQR)	9 (6, 13), n=57	14 (8, 20), n=51	<0.001
Carbapenem therapy	35 (41)	43 (72)	0.0002
Duration of use, days, median (IQR)	5 (2, 11)	8 (5, 14)	0.01
Gram-positive active agent use	54 (63)	51 (85)	0.003
Duration of use, days, median (IQR)	4.5 (2, 10)	6 (4, 11)	0.12
Anaerobic-active agent use	33 (38)	22 (37)	0.83
Duration of use, days, median (IQR)	8 (4, 13)	9.5 (4, 18)	0.41
Concomitant Medications			
Acid-suppressing medication use	69 (80)	55 (92)	0.06
Duration of use, days, median (IQR)	10 (5, 26)	14 (9, 23)	0.09
Immunosuppressant use	34 (40)	19 (32)	0.33
Duration of use, days, median (IQR)	5 (2, 10)	6 (2, 14)	0.47

### Clinical outcomes

Coinfected patients took almost twice as long to achieve clinical stability compared to the CRKP-only group, but the difference was not statistically significant (median 7 days vs. 4.5 days, p=0.11) (**Table 4**). Notably, both overall and post-infection median length of stay was significantly prolonged in the coinfected group (overall: median 15 days vs. 10 days, p<0.01; post-infection: 14 days vs 8.5 days, p=0.0003). In-hospital mortality occurred in 17% of patients with similar rates between study groups. Among those discharged, a trend towards shorter time to readmission was observed for the coinfected group compared to CRKP-only group (median 27 vs 41 days, p=0.13) (**Table 4**).

**Table 4:** Clinical Outcomes.

	CRKP-Only n=86 (%)	Coinfected n=60 (%)	p-value
Reached clinical stability, n=80 vs. 60	51 (64)	33 (55)	0.30
Time to clinical stability, median days (IQR)	4 (1, 8)	7 (2, 11)	0.16
Overall length of stay, median days (IQR)	10 (5, 20)	15 (10, 24)	<0.01
Post-infection length of stay, median days (IQR)	8.5 (4.0, 13.0)	14.0 (8.0, 21.5)	0.0003
In-hospital mortality	14 (16)	11(18)	0.75
Discharge status (Survivors: n=72 vs. 49)*			0.14
Lower level	1 (1)	4 (8)	
Same level	61 (85)	36 (74)	
Higher level	10 (14)	9 (18)	
Readmission, any (Survivors: n=72 vs. 49)	35 (49)	23 (47)	0.86
Readmission for infection (Survivors: n=72 vs. 49)	30 (42)	22 (45)	0.72
Interval between date of discharge and first readmission, days, median (IQR)	41 (14, 147)	27 (13, 46)	0.13

* Lower level discharge status included patients discharged from OSH to SNF/LTCF or rehab, and from SNF/LTCF to home or rehab; Same level included from home to home, OSH to OSH and SNF/LTCF to SNF/LTCF; Higher level included from home to OSH or SNF/LTCF, from OSH to hospice, and from SNF/LTCF to OSH or hospice. Abbreviation: OSH=outside hospital, SNF/LTCF=skilled nursing facility/long-term care facility.

### Multivariate analysis

**Figure 1:**
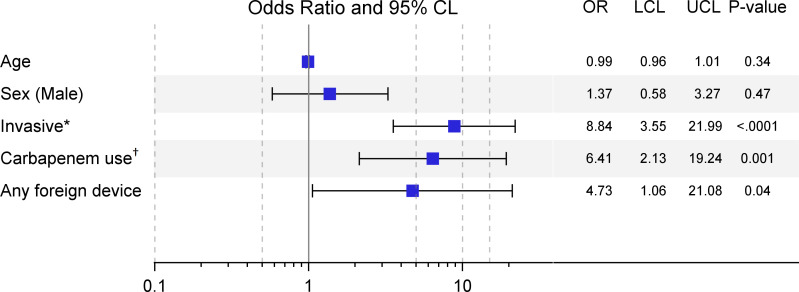
Multivariable models for risk factors significantly associated with coinfection (N=146). *Invasive infections include bacteremia or pneumonia (noninvasive were infections involving urinary tract or wound sites).†Carbapenem use within 90 days prior to admission; foreign device includes Foley catheter, gastric or nasogastric feeding tube, or mechanical ventilation.

By multivariable logistic regression analysis, the following clinical variables were identified for our cohort as independent risk factors that were significantly associated with coinfection after controlling for age and sex: invasive infection (OR 8.84, 95% CI 3.55-21.99, p<0.0001), carbapenem use within 90 days prior (OR 6.40, 95% CI 2.13-19.24, p=0.001), and any foreign device use prior or during hospital admission (OR 4.73, 95% CI 1.06-21.08, p=0.04) (**Figure 1**).

## Discussion

In this descriptive analysis, we sought to identify risk factors present on index hospital admission that differentiates patients with CRKP monoinfection from patients coinfected with other carbapenem-resistant organisms, namely *Pseudomonas aeruginosa* and *Acinetobacter baumannii* and associated outcomes. To date, knowledge about risk factors for CRKP coinfection with CRPA/CRAB and its subsequent impact on morbidity and mortality remains limited. *Marchaim et al.* previously demonstrated that patients co-colonized with CRE and CRPA/CRAB were older and more severely ill, as reflected by increased healthcare exposure (including more antimicrobial exposure prior to positive CRKP culture), more underlying chronic diseases, more invasive infections, and requirement for ICU admission at time of CRE isolation (Marchaim et al., 2012). Importantly, the authors identified co-colonization as an independent predictor for 90-day mortality. *Mammina et al.* described the epidemiology of patients co-colonized with CRKP and CRAB in two ICUs in Italy. Interestingly, in that study, factors such as younger age, major trauma (as the primary diagnosis for admission), and length of stay were positively associated with co-colonization (Mammina et al., 2013). No significant differences in antimicrobial exposure within 30 days of admission or mortality were observed between the two groups, although it should be noted that the study investigated the contribution of CRKP as a co-colonizer rather than the primary pathogen of interest whereby the “not co-colonized” group consisted of patients with CRAB only.

Our study cohort of CRKP-infected patients is comprised of an elderly population in whom the majority were admitted from the skilled nursing or long-term care facilities. Consistent with published studies examining co-colonized patients, we found that coinfected patients were severely debilitated with most lacking functional status on all activities of daily living particularly with continence and feeding. A majority of coinfected patients required a chronic Foley catheter and nearly all required a feeding tube. In addition, coinfected patients were significantly more likely to have had pneumonia in the past year which may explain the observation that the respiratory tract was the predominant site of infection for the coinfected group whereas the urinary tract was the predominant site for the CRKP-only group. Importantly, prior pneumonia likely reduced the lung physiologic capacity to mount an adequate response against opportunistic pathogens such as *Pseudomonas aeruginosa* as evidenced by twice as many coinfected patients requiring ICU admission and three-fold greater number of patients requiring mechanical ventilation compared to patients infected with only CRKP.

Moreover, coinfected patients were more likely to have had antibiotic exposure within 90 days prior to admission, particularly to carbapenems, followed by beta-lactam/beta-lactamase inhibitor, parenteral vancomycin, and tigecycline. Others have also shown that glycopeptide administration within the previous three months was significantly higher among coinfected patients (Marchaim et al., 2012). The complex underlying medical conditions of our patients likely required the use of broad spectrum agents to provide adequate coverage but also predisposing them to infections caused by multidrug-resistant organisms. Specifically, carbapenem overuse is of particular concern, whereby overexposure may select for carbapenem-resistant organisms through different mechanisms such as porin deletion or mutation, efflux pump overexpression, and upregulation of carbapenem resistance genes such as *bla*_KPC_ (Park et al., 2011; Voor in 't holt et al., 2014; Palavutitotai et al., 2018; Tsao et al., 2018; Zou et al., 2018). By multivariate analysis, clinical features identified to be significantly associated with coinfection in our study cohort were invasive infections, use of any medical device, and prior carbapenem exposure.

This study assessed the antimicrobial regimens utilized to treat monoinfected vs. coinfected patients. It is interesting that an ineffective empiric regimen was initiated more frequently among patients in the CRKP-only than the co-infected group. This difference may be due to the more prominent history of prior isolation of ESBL and carbapenem-resistant organisms (CRO), higher rate of recent antibiotic exposure, and higher severity of infection thereby necessitating the initiation of broad-spectrum last-line antibiotics among the coinfected group. Additionally, the significantly greater frequency of prior isolation of multi-drug resistant organisms among coinfected patients compared to the CRKP-only group provides prescribers with knowledge of antibiotic sensitivities *a priori* through previous culture and susceptibility information to help guide empiric treatment choices. Nonetheless, ineffective empiric therapy remained unacceptably high in both groups. Our findings underscore the need to consider broad spectrum agents that have reliable activity against both CRKP and CRPA for empiric therapy in at-risk populations for coinfection (e.g. debilitated patients admitted with pneumonia from a skilled nursing or long term care facility with a history of carbapenem-resistant pathogen isolation).

Of interest, we observed a trend towards increased and prolonged usage of acid suppressing medications, specifically proton-pump inhibitors (PPIs) or histamine_2_ receptor antagonists (H2RAs), among coinfected patients. Our findings are consistent with that of a 2013 nested case-control study investigating factors for CRE acquisition, where univariate analysis reported that patients who acquired CRE during hospitalization were 2.5 times more likely to receive gastric acid suppressing medications compared to matched controls (p=0.01) (Swaminathan et al., 2013). Numerous investigations have shown that acid-suppressing medication use, especially proton-pump inhibitors, is associated with increased risk of hospital-acquired infections, including bacterial pneumonia (Herzig et al., 2009; Eom et al., 2011). The underlying mechanism for this correlation is unclear but is likely multifactorial. Inhibition of gastric acid secretion may allow for bacterial overgrowth and colonization in the upper alimentary tract with subsequent translocation to the respiratory tract via aspiration (Laheij et al., 2004). Use of PPIs may also alter seromucinous secretions and encourage bacterial growth (Savarino et al., 2009), and *in vitro* studies have demonstrated that acid suppression may impair neutrophil and natural killer cell function (Zedtwitz-liebenstein et al., 2002; Kedika et al., 2009). Our findings suggest that careful considerations should be taken when considering prophylactic use of PPI/H2RA in hospitalized patients, as acid suppression may have detrimental implications for acquiring nosocomial infection.

Since we have previously reported that up to one-third of patients hospitalized with non-bacteremic CRKP infections were readmitted within 30 days of hospital discharge (Ny et al., 2015), we sought to evaluate disposition of patients between care settings as well as in-hospital mortality and readmissions. Overall, the majority of patients (75%) were admitted from either skilled nursing or long-term care facilities (SNF/LTCF); 88% of whom were discharged back to SNF/LTCF. Among those who were admitted from home, coinfected patients were more likely to be discharged to a SNF/LTCF than CRKP-only group (50%, 3/6 vs 26%, 5/19), though the small sample size should be interpreted with caution. Similarly, we observed a trend towards shorter time to first readmission in the coinfected group. In contrast to findings from *Marchaim et al.*, coinfection was not associated with increased mortality in our cohort. While mortality differences were not observed, coinfected patients demonstrated a significantly greater burden of care, as evidenced by longer length of hospital stay and higher need for foreign device use over a longer duration during hospitalization.

Our study had several limitations, including the retrospective nature of the study design as well as a relatively small sample size. The diagnosis of infection may be subjected to prescriber bias, especially among the coinfected patients with a high proportion of ESBL and CRO history and high utilization of foreign devices. Signs and symptoms commonly associated with infection such as fever and vital sign instability due to a non-infectious etiology (e.g., central or drug fevers, suboptimal use/placement of foreign device, drug-induced bradycardia or tachycardia) could be misinterpreted as early signs of infection especially in the setting of a positive culture. Additionally, knowledge of prior culture and sensitivity information is likely a confounding variable that could affect timing of antibiotic initiation and selection, thereby favoring outcome in the co-infected group. The ability to capture data pertinent to outside hospitalizations such as antimicrobial administration as well as readmissions to outside institutions also pose limitations to the completeness of data collection.

## Conclusion

Taken together, we observed that coinfection with CRKP and another carbapenem-resistant pathogen significantly increased morbidity and healthcare burden. Negative impact on in-hospital resource utilization may be attributable to greater need for ICU admission, prolonged hospitalization, and short interval (< 30 day) from discharge to readmission. In this elderly cohort with severe debilitation and complex underlying medical conditions, austere measures that include the judicial use of antimicrobial and acid-suppressing agents, rigorous infection prevention practice as well as regular assessment of indication and duration of foreign device (feeding tube, catheter) placement are needed to disrupt the vicious cycle of repeated infections involving multiple carbapenem-resistant pathogens and intra- and interfacility spread.

## Data availability statement

The raw data supporting the conclusions of this article will be made available by the authors, without undue reservation.

## Ethics statement

The studies involving humans were approved by Huntington Hospital Advarra IRB: Pro00036497. The studies were conducted in accordance with the local legislation and institutional requirements. The ethics committee/institutional review board waived the requirement of written informed consent for participation from the participants or the participants’ legal guardians/next of kin because this was a retrospective descriptive analysis.

## Author contributions

CK and AW-B designed the study. CK gathered data and wrote the first draft of the manuscript. CK, AS, AW-B, and ML participated in data interpretation. AS, AW-B, and ML modified and revised the manuscript critically for intellectual content. All authors contributed to the article and approved the submitted version.
